# Robotic-Arm Assisted Technology’s Impact on Knee Arthroplasty and Associated Healthcare Costs

**DOI:** 10.36469/001c.37024

**Published:** 2022-08-23

**Authors:** David J. Kolessar, Daniel S. Hayes, Jennifer L. Harding, Ravi T. Rudraraju, Jove H. Graham

**Affiliations:** 1 Geisinger Health System, Danville, Pennsylvania

**Keywords:** orthopedics, knee arthroplasty, unicompartmental, economic, robotic arm-assisted surgery

## Abstract

**Background:** The number of total knee arthroplasties (TKA) carried out globally is expected to substantially rise in the coming decades. Consequently, focus has been increasing on improving surgical techniques and minimizing expenses. Robotic arm–assisted knee arthroplasty has garnered interest to reduce surgical errors and improve precision.

**Objectives:** Our primary aim was to compare the episode-of-care cost up to 90 days for unicompartmental knee arthroplasty (UKA) and TKA performed before and after the introduction of robotic arm–assisted technology. The secondary aim was to compare the volume of UKA vs TKA.

**Methods:** This was a retrospective study design at a single healthcare system. For the cost analysis, we excluded patients with bilateral knee arthroplasty, body mass index >40, postoperative infection, or noninstitutional health plan insurance. Costs were obtained through an integrated billing system and affiliated institutional insurance company.

**Results:** Knee arthroplasty volume increased 28% after the introduction of robotic-assisted technology. The TKA volume increased by 17%, while the UKA volume increased 190%. Post introduction, 97% of UKA cases used robotic arm–assisted technology. The cost analysis included 178 patients (manual UKA, n = 6; robotic UKA, n = 19; manual TKA, n = 58, robotic TKA, n = 85). Robotic arm–assisted TKA and UKA were less costly in terms of patient room and operating room costs but had higher imaging, recovery room, anesthesia, and supply costs. Overall, the perioperative costs were higher for robotic UKA and TKA. Postoperative costs were lower for robotic arm–assisted surgeries, and patients used less home health and home rehabilitation.

**Discussion:** Surgeons performed higher volumes of UKA, and UKA comprised a greater percentage of total surgical volume after the introduction of this technology. The selective cost analysis indicated robotic arm–assisted technology is less expensive in several cost categories but overall more expensive by up to $550 due to higher cost categories including supplies and recovery room.

**Conclusions:** Our findings show a change in surgeons’ practice to include increased incidence and volume of UKA procedures and highlights several cost-saving categories through the use of robotic arm–assisted technology. Overall, robotic arm–assisted knee arthroplasty cost more than manual techniques at our institution. This analysis will help optimize costs in the future.

## BACKGROUND

The demand for total knee arthroplasty (TKA) has grown dramatically in the past 2 decades, and recent projection models estimate TKA volume to reach 1.5 million to 1.7 million cases per year by 2050.^1,2^ Knee arthroplasty (KA) is associated with substantial expenditures and rising healthcare spending across the United States.^3–5^ As surgical demand has risen, so has the economic impact of these procedures despite enhancements in implant quality, operative techniques, rehabilitation, and complication management to reduce expenditures and improve quality of care.^6–8^ Over the past decade, robotic arm–assisted knee arthroplasty (rKA) has risen in popularity as an avenue to enhance operative efficiency, improve outcomes, and decrease postoperative costs.^8,9^

The use of rKA is associated with improved clinical, radiographic, and patient-reported outcomes. Advances in rKA technology have led to increased surgical accuracy and precision, improved component alignment, and better soft-tissue protection when compared with manual knee arthroplasty (mKA).^10–14^ In a large meta-analysis of robotic arm–assisted TKA (rTKA) and manual TKA (mTKA), Onggo et al^15^ found superior precision of prosthesis implantation in rTKA but no difference in clinical outcomes or complication profiles. Postoperatively, patients who underwent rKA have demonstrated lower pain scores and improved physical function and have reported improved satisfaction and activity when compared with mKA patients.^11,16,17^ Patients with rKA surgery may require less inpatient therapy, experience faster hospital discharge times, and utilize less postoperative therapy, all of which are major contributors to healthcare costs.^8,9,18^

Despite the many advantages of rKA, early adoption of this technology has been slow with varied rates of utilization influenced by practice type, surgical volume, clinical concerns, and financial considerations.^19,20^ The gradual increase in utilization of rKA has been attributed to the considerable initial capital equipment investment, additional preoperative imaging costs, lack of long-term outcome studies, and concerns about an associated learning curve.^8,19^ Several studies examining rKA training have demonstrated a short learning curve, requiring only a handful of cases to integrate into the preoperative and surgical workflow. There was no learning curve effect on implant positioning and only a brief time needed to achieve similar operative times between rTKA and mTKA procedures.^21–24^

The use of robotic arm–assisted technology in unicompartmental knee arthroplasty (rUKA) has decreased postoperative complications and reoperation rates compared with manual UKA (mUKA).^25,26^ High reoperation rates have historically been problematic with mUKA.^27,28^ The use of robotic arm–assisted technology for this technically challenging procedure yields more desirable clinical outcomes and may serve as a cost-effective procedure when accounting for the reduction in complications.^29,30^ Robotic arm–assisted KA may be beneficial in providing healthcare cost savings by increasing UKA over mTKA and rTKA for appropriate UKA candidates; however, no studies have reported a cost analysis and surgeon practice pattern variation following the introduction of robotic-arm assistance technology.

With the introduction of new operative technologies such as rKA, it is imperative to perform economic and cost-effectiveness research. Studying the financial impact of rKA compared with mKA is a critical exercise in determining the relative advantages that robotic technology may afford. Previous research on the economic impact of robotic arm–assisted technology is not without limitations. Prior economic analyses have utilized costs obtained from Medicare 100% Analytical Files, allowable amounts from private health plans, and Medicare Inpatient Prospective Payment data.^9,29,31–33^ Payments obtained from these commercial and administrative databases were unable to determine claim costs and allowed amounts paid vs true hospital facility costs. Previous studies did not include the cost of a preoperative computed tomographic (CT) scan, which is required for creating a computerized 3-dimensional virtual knee image used during preoperative planning and the surgical procedure.^9,32,33^ There remains a paucity of information regarding the influence of robotic arm–assisted technology on arthroplasty volume and practice pattern change, and no studies have examined the economic benefit of UKA and TKA volume following introduction of this technology.

Approximately a third of patients in our integrated health system are insured by its affiliated health plan, providing an ideal environment for studies examining the overall costs of care. The purpose of this study is to examine the influence of the introduction of robotic arm–assisted technology on surgeon volumes of UKA and TKA and to compare both perioperative (preoperative and in-hospital) episode-of-care costs and postoperative (out-of-hospital) health care costs and utilization for UKA and TKA with and without robotic arm–assisted technology.

## METHODS

This study was reviewed and approved by the health system’s institutional review board. Electronic medical records (Epic, Epic Systems Corporation, Verona, Wisconsin) and health plan databases were reviewed to identify patients who underwent UKA or TKA by using *Current Procedural Terminology* (CPT) codes 27446 and 27447. The retrospective review interval was July 2016 through September 2019. Cases performed by low-volume surgeons, defined as surgeons with fewer than 10 KA procedures, were excluded. Surgeons who did not use rKA or who were hired or departed the health system during the study period were also excluded. Patients were excluded if they had prior KA on the same knee, simultaneous or staged bilateral KA, inflammatory arthritis, body mass index greater than 40 kg/m^2^, active infection, or suspected latent infection in or about the joint. Robotic arm–assisted KA became available at 2 of our 9 hospital campuses on January 3, 2018, and 18-month volumes before (July 1, 2016–December 31, 2017) and after (April 1, 2018–September 30, 2019) this date were compared at these 2 locations, excluding a 3-month learning curve period immediately after introduction. We compared how introduction of the Mako robotic arm–assisted technology (Mako Surgical, Stryker Corp, Fort Lauderdale, Florida) affected surgeon volumes and ratio of TKA to UKA surgeries performed.

Second, cost analysis was performed on all patients with the health system’s integrated insurance plan as primary coverage who underwent TKA or UKA surgery during the study period at one of the robotic-equipped hospitals. Costs were categorized both by timing (ie, perioperative vs postoperative ≤90 days) and by type (ie, imaging, laboratory, anesthesia, physician fees, implant devices, supplies, prescriptions, room charges, therapy/rehabilitation, home health care). In terms of perioperative costs (**Table 1**), implant costs and associated supplies (ie, femoral, tibial, and patellar components; cement; mixing equipment) were removed from the analysis as these cost variables were related to individual surgeon preference rather than manual vs robotic technology. Perioperative costs reflect actual care costs recorded by the health system at the point of service, whether the procedure was done in an inpatient or outpatient setting. As patients may have sought care outside of the health system after discharge, postoperative costs represent the actual total amounts allowed by the health plan and paid, by both insurer and patient. This allows for detailed granular analysis of actual perioperative costs to the health system, while also capturing postoperative expenses accrued internal and external to the health system. Postoperative health care utilization includes total number of home health, home rehabilitation, and outpatient rehabilitation visits. Discharges to skilled nursing facilities (<2% of patients) and readmissions costs were included in the total postoperative cost category. Medication costs were eliminated from both perioperative and postoperative costs due to the possibility of overlap and dependence on patient preexisting comorbidities.

**Table 1.** Perioperative Cost Definitions

**Cost Category**	**Definition**
Anesthesia	Cost of anesthesia and associated labor. Common anesthesia costs include nerve injections, blocks, and ultrasound guidance if required. Associated labor may include time of anesthesia (by time period, eg, 90-150 min, 151-210 min, etc), CRNA, and supervision.
Imaging	Cost of all imaging procedures and labor during the perioperative period. Common costs include x-rays and interpretation of results if specified.
Implant	Cost of implant knee system and associated materials (femoral, tibial, and patellar components; cement; mixing equipment and devices).
Lab	Cost of all lab work ordered during the perioperative period. Common lab orders include metabolic panels, blood typing, CBC, hemoglobin, coagulation studies, and EKG.
Operating room	Institutional cost associated with use of operating room assessed every 15 minutes.
Other perioperative	Includes costs not directly tied to other categories, often unique and not seen often. Examples may include ventilation management and neuromuscular repeat patient education.
Physician	Cost of hospitalists and specialists other than the orthopedic surgeon. Consults are categorized by level (1-4).
Preoperative CT scan	Cost of CT scan during a preoperative office visit for robotic-assisted surgery patients
Preoperative prep	Cost associated with preoperative prep of the room and patient. Costs are assessed every 15 minutes.
Prescriptions	Cost of ordered and prescribed medications.
Recovery	Cost associated with patient recovery after surgery prior to transfer. Assessed every 30 minutes.
Room	Institutional cost associated with use of a hospital room.
Supply	Costs associated with supplies used during the operation and recovery process (usually single-use supplies). Common costs include saw blades, drill bits, TED stockings, gauze, blankets, gowns, and replacement filters.
Surgeon	Cost associated with the orthopedic surgery team performing the operation.
Therapy	Cost associated with therapeutic activity and procedures assessed every 15 minutes. Common costs include assessment of mobility, discharge goal status, occupational and physical therapy evaluations, and self-care training/status.

Abbreviations: CBC, complete blood cell count; CRNA, certified registered nurse anesthetist; CT computed tomography; EKG, electrocardiogram.

### Statistical Analysis

We used descriptive statistics only to examine the volume of cases in the study period. For the cost analysis, covariates (eg, sex, age, body mass index, laterality) between the 4 groups (robotic and manual UKA and TKA) were first compared to examine potential for confounding, with simple descriptive statistics (eg, means, percentages) and standardized differences. Generalized linear regression modeling was then used to compare mean outcomes (costs, number of visits, and length of stay) between the 2 pairs of groups. For costs, a log-link function and gamma error distribution was used. For counts (eg, number of visits, length of stay), a log-link function and either a Poisson or negative-binomial distribution was used, possibly with zero inflation if there were a large number of cases with counts of zero. All analysis were done using SAS (SAS 9.4, SAS Institute, Cary, North Carolina) or R 4.0.0 (The R Group, Vienna, Austria) statistical software, with contrasts of *P* < .05 considered statistically significant.

## RESULTS

The overall KA case volume for the 18 months following the introduction of robotics increased 27.9%, from 595 to 761 cases. The TKA volume increased by 16.9% (557 to 651 cases), while the UKA volume increased 189.5% (38 to 110 cases). The ratio of TKA to UKA before and after robotic availability narrowed from 15:1 to 6:1, and UKA increased from 6.4% to 14.5% of all KAs performed. **Table 2** displays how each included surgeon’s practice pattern changed after robotic arm–assisted surgery was introduced.

**Table 2.** Surgeon UKA vs TKA Volumes Before and After Introduction of Robotic Arm–Assisted Technology

	**Years of Experience**	**18 Months Before Introduction (7/1/2016-12/31/2017)**		**18 Months After Introduction (4/1/2018-9/30/2019)**				
		**mUKA**	**mTKA**	**mUKA**	**mTKA**	**rUKA**	**rTKA**	**% Increase**
Surgeon A	17	13	106	2	42	26	77	—
Surgeon B	25	9	188	0	143	16	99	
Surgeon C	6	16	200	1	124	52	62	
Surgeon D	23	0	63	0	95	13	9	
Total UKA volume		38	—	3	—	107	—	189.50
Total TKA volume		—	557	—	404	—	247	16.90
Total KA volume		595			761			27.90
TKA-UKA ratio		557:38 (15:1)			651:110 (6:1)			

Abbreviations: KA, knee arthroplasty; mTKA, manual total knee arthroplasty; manual unicompartmental knee arthroplasty; rTKA, robotic arm–assisted total knee arthroplasty; rUKA, robotic arm–assisted unicompartmental knee arthroplasty; TKA, total knee arthroplasty; UKA, unicompartmental knee arthroplasty.

**Table 3** compares the baseline characteristics between manual and robotic cohorts, separately for UKA and TKA, with standardized differences |d| > 0.10 indicating poor balance between the groups. There are some areas in which the groups are not balanced, although the magnitude of those differences are small.

**Table 3.** Baseline Characteristics of Patients in the 4 Groups (UKA vs TKA, Manual vs Robotic)

	**UKA**			**TKA**		
	**mUKA (n=6)**	**rUKA (n=29)**	**|d|**	**mTKA (n=58)**	**rTKA (n=85)**	**|d|**
Sex, n (%)						
Female	2 (33)	16 (55)	**0.45**	31 (53)	44 (52)	0.03
Male	4 (67)	13 (45)		27 (47)	41 (4)	
Age at surgery (y)						
Mean (SD)	56 (5)	60 (9)	**0.55**	66 (10)	62 (9)	**0.42**
Range	(49, 61)	(48, 80)		(44, 88)	(43, 81)	
Race, n (%)						
White	6 (100)	29 (10)	0.00	58 (100)	83 (98)	**0.22**
Asian	0 (0 )	0 (0 )	0.00	0 (0 )	1 (1 )	**0.15**
Black or African American	0 (0 )	0 (0 )	0.00	0 (0 )	1 (1 )	**0.15**
Hispanic or Latino, n (%)			0.00			
Yes	0 (0)	0 (0)	0.00	1 (2)	0 (0)	**0.19**
No	6 (100)	29 (100)		57 (98)	85 (100)	
ASA score, n (%)						
1	0 (0)	1 (3)	**0.27**	1 (2)	1 (1)	0.05
2	1 (17)	12 (41)	**0.57**	39 (67)	48 (56)	**0.22**
3	5 (83)	16 (55)	**0.64**	17 (29)	35 (41)	**0.25**
4	0 (0)	0 (0)	0.00	1 (2)	1 (1)	0.05
Surgeon, n (%)						
A	0 (0)	5 (17)	**0.65**	0 (0)	10 (12)	**0.52**
B	3 (50)	7 (24)	**0.56**	22 (38)	34 (40)	0.04
C	2 (33)	6 (21)	**0.29**	22 (38)	21 (25)	**0.29**
D	1 (17)	11 (38)	**0.49**	14 (24)	20 (24)	0.01
Laterality, n (%)						
Left	2 (33)	17 (59)	**0.52**	31 (53)	30 (35)	**0.37**
Right	4 (67)	12 (41)		27 (47)	55 (65)	
Hospital, n (%)						
GSACH	3 (50)	12 (41)	**0.17**	22 (38)	44 (52)	**0.28**
GWV	3 (50)	17 (59)		36 (62)	41 (48)	
Setting, n (%)						
Inpatient	2 (33)	4 (14)	**0.47**	52 (90)	49 (58)	**0.78**
Outpatient	4 (67)	25 (86)		6 (10)	36 (42)	
Charlson Comorbidity Index, mean (SD)	1.7 (1.0)	2.0 (1.5)	**0.59**	3.1 (1.7)	2.8 (1.6)	**0.18**
BMI category, n (%)						
<30	0 (0)	10 (34)	**1.03**	23 (40)	32 (38)	0.04
≥30	6 (100)	19 (66)		35 (60)	53 (62)	

Abbreviations: ASA, American Society of Anesthesiologists Physical Status Classification System; BMI, body mass index; GSACH, Geisinger Shamokin Area Community Hospital, GWV, Geisinger Wyoming Valley Medical Center; mTKA, manual total knee arthroplasty; manual unicompartmental knee arthroplasty; rTKA, robotic arm–assisted total knee arthroplasty; rUKA, robotic arm–assisted unicompartmental knee arthroplasty; TKA, total knee arthroplasty; UKA, unicompartmental knee arthroplasty.

Standardized difference scores |d| of greater than 0.10 are denoted in bold.

**Table 4** shows the perioperative cost comparisons between manual vs robotic for both UKA and TKA. Mean length of stay (LOS) in days was approximately equal for all groups, yet rUKA and rTKA showed wider ranges in LOS than mUKA and mTKA (2.9 and 3.8 vs 1.1 and 2.2 days, respectively). As expected, only the robotic patients had any preoperative CT scan costs. A significantly larger proportion of manual patients had any inpatient room costs than robotic patients, for both UKA (67% vs 14%, *P* = .01) and TKA (90% vs 58%, *P* < .001). Five patients (2.8%) were discharged on their day of surgery, with all 5 patients undergoing robotic arm–assisted surgery (**Table 4**). However, this translated into significantly higher mean inpatient room costs only for the mUKA patients (mUKA $281 vs rUKA $82, *P* = .04). For UKA, a greater proportion of robotic patients had any physician costs significantly higher than the manual patients did (69% vs 17%, *P* = .03), but this did not translate into higher mean physician costs per patient overall.

**Table 4.** Comparisons of Perioperative Costs for Manual vs Robotic KA Patients

		**UKA**			**TKA**	
**Cost**	**mUKA (n=6)**	**rUKA (n=29)**	***P* Value**	**mTKA (n=58)**	**rTKA (n=85)**	***P* Value**
Anesthesia						
Mean (SD)	523 (52)	586 (111)	.14	628 (90)	714 (103)	<.0001^b^
Range	(435, 879)	(416, 861)		(427, 825)	(530, 1104)	
Imaging						
Mean (SD)	26 (2)	40 (52)	.12	30 (7)	42 (59)	.0003^b^
Range	(24, 28)	(24, 306)		(24, 66)	(16, 439)	
Labs						
Mean (SD)	45 (18)	36 (46)	.55	49 (36)	65 (69)	.06
Range	(30, 72)	(2, 255)		(1, 216)	(1, 408)	
Operating room						
Mean (SD)	610 (258)	368 (129)	.002^b^	403 (174)	354 (124)	.05
Range	(194, 936)	(210, 674)		(163, 805)	(163, 713)	
Other inpatient						
Mean (SD)	305 (323)	363 (252)	.66	415 (280)	361 (247)	.27
Range	(27, 754)	(43, 670)		(27, 1059)	(34, 753)	
Physician						
Patients with any, n (%)	1 (1)	20 (69)	.03^a^	32 (5)	54 (64)	0.38
Mean (SD)	15 (38)	60 (57)	.93	75 (91)	72 (86)	0.33
Range	(0, 93)	(0, 167)		(0, 274)	(0, 318)	
Preoperative CT						
Patients with any, n (%)	0 (0)	29 (100)		0 (0)	85 (100)	
Mean (SD)	—	114 (22)	<.0001^b^	—	115 (23)	<.0001^b^
Range	—	(74, 157)		—	(74, 165)	
Preopertive prep						
Mean (SD)	446 (287)	400 (302)	.75	400 (307)	494 (328)	.15
Range	(170, 728)	(87, 849)		(86, 901)	(86, 1001)	
Recovery						
Mean (SD)	226 (205)	613 (375)	.008^a^	258 (434)	602 (724)	<.0001^b^
Range	(70, 625)	(65, 1605)		(42, 1836)	(28, 2629)	
Room						
Patients with any, n (%)	4 (67)	4 (14%)		52 (90)	49 (58)	
Mean (SD)	281 (218)	82 (217)	.02^a^	725 (593)	531 (677)	<.0001^b^
Range	(0, 436)	(0, 847)	.04^a^	(0, 3390)	(0, 2542)	.29
Supplies						
Mean (SD)	651 (176)	1161 (384)	<.0001^b^	868 (518)	1042 (525)	.03^a^
Range	(435, 850)	(713, 2034)		(406, 3340)	(456, 2130)	
Therapy						
Mean (SD)	160 (44)	201 (76)	.24	235 (103)	287 (126)	**.**01^b^
Range	(121, 235)	(28, 370)		(86, 442)	(71, 663)	
Total perioperative costs						
Mean (SD)	3287 (271)	4025 (489)	.0001^b^	4087 (1085)	4668 (644)	<.0001^b^
Range	(3002, 3632)	(2674, 4719)		(2745, 7903)	(3330, 6376)	
Length of stay (days)						
Mean (SD)	1.6 (0.5)	1.4 (0.6)	.51	1.8 (0.6)	1.8 (0.8)	.94
Range	(1.2, 2.3)	(0.4, 3.3)		(1.1, 3.3)	(0.4, 4.2)	
Same-day discharge, n (%)	0 (0.0)	3 (10.3)		0 (0.0)	2 (2.4)	

Abbreviations: mTKA, manual total knee arthroplasty; manual unicompartmental knee arthroplasty; rTKA, robotic arm–assisted total knee arthroplasty; rUKA, robotic arm–assisted unicompartmental knee arthroplasty; TKA, total knee arthroplasty; UKA, unicompartmental knee arthroplasty.

^a^*P* < .05.

^b^*P* < .001.

The robotic group had lower mean OR costs than the manual group for both UKA ($368 vs $610, *P* = .002) and TKA ($354 vs $403, *P* = .05). The robotic TKA group had mean inpatient therapy costs that were significantly higher than the manual TKA group, although the actual difference was only $52 per patient ($287 vs $235, *P* = .01). Robotic patients also had significantly higher mean recovery costs than manual patients, for both UKA ($613 vs $226, *P* = .008) and TKA ($602 vs $258, *P* < .0001). The rTKA group had significantly higher mean imaging costs than the mTKA group, although again the magnitude of the difference per patient was small ($42 vs $30, *P* = .0003). The rTKA group had significantly higher mean anesthesia costs than the mTKA group ($714 vs $628, *P* < .0001). The robotic groups had significantly higher mean supply costs than the manual groups for both UKA ($1161 vs $651, *P* < .001) and TKA ($1042 vs $868, *P* = .03). There were no significant differences with respect to lab costs, other perioperative costs, or preoperative prep costs. However, for both UKA and TKA, the total perioperative cost was statistically significantly higher for robotic than manual KA patients. For UKA, this difference in means was $4025 vs $3287 (*P* = .0001), and for TKA, $4668 vs $4087 (*P* < .0001). Note these figures do not represent the actual total cost of care, as some expenses were excluded, as mentioned previously.

**Table 5** compares postoperative (out-of-hospital) utilization (eg, home health, home rehab, and outpatient rehab visits) and costs between manual vs robotic, stratified by UKA and TKA. Robotic KA patients had significantly fewer home health visits than mKA patients (0.3 vs 1.8, *P* = .0001 for UKA, 0.1 vs 0.8, *P* < .0001 for TKA). Although the corresponding mean home health costs tend to be lower for rKA patients, these differences were not significant. Similarly, rKA patients had significantly fewer home rehab visits (2.0 vs 5.0, *P* = .0002 for UKA, 1.1 vs 3.9, *P* < .0001 for TKA) than manual patients. Again, the corresponding mean home rehab costs tend to be lower for rKA patients, but these differences were not significant. The only significant difference with respect to outpatient rehab visits were that rUKA patients had a higher mean number per patient than mUKA patients (9.9 vs 6.2, *P* = .004). There were no significant differences in outpatient rehab costs. Similarly, there were no significant differences with respect to total rehab costs and total postoperative costs, although these tend to be lower for robotic cases.

**Table 5.** Comparisons of Postoperative Utilization and Costs for Manual vs Robotic KA Patients

		**UKA**			**TKA**	
	**mUKA (n = 6)**	**rUKA (n = 29)**	***P* Value**	**mTKA (n = 58)**	**rTKA (n = 85)**	***P* Value**
Home health visits						
Patients with any, n (%)	5 (83)	5 (17)	0.004^a^	17 (29)	5 (6)	.0002^b^
Mean (SD)	1.8 (1.7)	0.3 (0.8)	0.0001^b^	0.8 (2.0)	0.1 (0.7)	<.0001^b^
Range	(0, 5)	(0, 4)		(0, 8)	(0, 6)	
Home health costs						
Patients with any, n (%)	5 (83)	4 (14)		16 (28)	5 (6)	.0005^b^
Mean (SD)	156 (188)	18 (74)	**.**002^a^	84 (261)	17 (98)	.96
Range	(0, 487)	(0, 390)	0.64	(0, 1280)	(0, 818)	
OP rehab visits						
Patients with any, n (%)	4 (67)	21 (72)	0.99	43 (74)	73 (86)	.09
Mean (SD)	6.2 (6.0)	9.9 (10.3)	**.**004^a^	11.7 (10.5)	12.8 (8.0)	.07
Range	(0, 16)	(0, 42)		(0, 40)	(0, 39)	
OP rehab costs						
Patients with any, n (%)	4 (67)	21 (72)	.99	43 (74)	72 (85)	.14
Mean (SD)	474 (556)	792 (1003)	.28	826 (779)	991 (878)	.73
Range	(0, 1461)	(0, 5013)		(0, 2922)	(0, 4756)	
Home rehab visits						
Patients with any, n (%)	5 (83)	9 (31)	.03^a^	31 (53)	14 (16)	.0001^b^
Mean (SD)	5 (4)	2.0 (3.2)	.0002^b^	3.9 (4.7)	1.1 (2.8)	<.0001^b^
Range	(0, 12)	(0, 9)		(0, 20)	(0, 12)	
Home rehab costs						
Patients with any, n (%)	5 (83)	9 (31)	.03^a^	31 (53)	14 (16)	<.0001^b^
Mean (SD)	609 (432)	241 (382)	.74	482 (598)	137 (331)	.66
Range	(0, 1266)	(0, 1078)		(0, 2700)	(0, 1412)	
Total rehab costs						
Patients with any, n (%)	6 (100)	26 (90)	.99	57 (98)	77 (91)	.08
Mean (SD)	1083 (593)	1033 (968)	.83	1308 (752)	1128 (852)	.55
Range	(405, 1914)	(0, 5012)		(0, 3527)	(0, 4756)	
Total postoperative costs						
Patients with any, n (%)	6 (100)	26 (90)	.99	57 (100)	77 (91)	.08
Mean (SD)	1239 (571)	1051 (968)	.75	1602 (1226)	1196 (974)	.10
Range	(405, 1932)	(0, 5013)		(0, 7322)	(0, 5306)	

Abbreviations: KA, knee arthroplasty; mTKA, manual total knee arthroplasty; manual unicompartmental knee arthroplasty; OP, outpatient; rTKA, robotic arm–assisted total knee arthroplasty; rUKA, robotic arm–assisted unicompartmental knee arthroplasty; TKA, total knee arthroplasty; UKA, unicompartmental knee arthroplasty.

^a^*P* < .05.

^b^*P* < .001.

Within a few of the cost categories such as patient room and operating room (OR), robotic surgery was less expensive than manual when comparing within UKA or TKA procedures. Other areas, however, showed higher mean costs for rTKA than mTKA (eg, inpatient therapy, recovery room, imaging, anesthesia, supplies). Total perioperative cost differences were significantly higher when robotics were used ($738 and $581 differences for UKA and TKA, respectively), but the postoperative (out-of-hospital) cost differences were nonsignificantly lower at $188 and $406 less for rUKA and rTKA, respectively. Summating our mean total perioperative and postoperative analyzed costs, mUKA had the lowest cost ($4526), while rUKA ($5076) was less costly than both manual ($5689) and robotic ($5846) TKA. These amounts do not represent total cost of care, as fixed costs were regarded as constants and excluded, as were implant costs.

## DISCUSSION

In this report, we saw a large increase in UKA procedures with robotic arm–assisted technology. Surgeons adopting this technology performed 189.5% more UKA procedures after introduction, with 97% of those being robotic arm–assisted procedures. Total perioperative cost comparison between robotic and manual UKA and TKA were statistically significantly higher for rKA patients. Postoperative utilization of home health and home rehab visits between robotic and manual UKA and TKA demonstrated that rKA patients had significantly fewer home health and home rehab visits. Despite these fewer visits, the corresponding lower mean home health cost and mean home rehab cost did not reach statistical significance. Although total postoperative costs were not significantly different, they tended to be lower on average for rKA patients. Our combined mean perioperative and postoperative costs for manual and robotic UKA and TKA revealed the lowest cost associated with mUKA, while rUKA was less costly than both manual and robotic TKA.

During our study period, there was an increase of overall KA surgical output of 27.9% (**Table 2**). A number of circumstances may be responsible for this arthroplasty volume increase. First, our institution was the only regional healthcare system to have rKA available during the study period. Second, marketing this technology may have fueled patient interest and subsequent pursuit of healthcare systems and surgeons with this capability. Although a national trend of TKA volume has been projected to increase over this decade, it does not explain our dramatic increase in UKA procedures.^34^

The ratio of TKA to UKA before and after Mako robotic arm–assisted technology availability narrowed from 15:1 to 6:1, and UKA procedures increased from 6.4% to 14.5% of all KAs performed (**Table 2**). Although this increase seems extreme, the 6:1 ratio of TKA to UKA aligns with the MANUKA study using “appropriate use criteria” developed by the American Academy of Orthopedic Surgeons based on both radiographic and clinical criteria, suggesting UKA is appropriate in approximately 14% of patients.^35,36^ Our increase in UKA procedures was coincident with the introduction of Mako technology. The preceding low utilization of UKA may be attributed to high technical difficulty of manual UKA, concerns of osteoarthritis progression, high revision rates, and challenges reported with revising UKA to TKA.^27,28,37–44^ One would anticipate exposure and availability to newer technology combined with institutional enthusiasm to stimulate trial interest, which occurred, yet not all surgeons adopted this resource. The use of robotic arm–assisted technology has been shown to improve UKA outcomes by limiting unforgiving surgical errors and optimizing accuracy of bony resections and implant placement.^45,46^ Unicompartmental KA has also demonstrated more rapid rehabilitation, greater range of motion, lower infection rates, and shorter hospital stays compared with primary TKA, all of which may decrease surgeon apprehension to perform UKA.^19,39–43,45,46^ Additionally, surgical indications have broadened to include select patients with anterior knee pain, patellofemoral arthritis, asymptomatic lateral meniscal pathology, obesity, and older patients, thus expanding the pool of potential candidates.^30,47,48^ This information, combined with more recent attractive mid-term survivorship data for UKA of 96.7% and 98.4% at 5 years by Park and Burger et al, respectively, are significant improvements on past UKA results, lending further support for increased utilization of UKA in properly selected patients.^27,48,49^ Encouraging survivorship data were recently published in a randomized controlled trial of robotic arm–assisted vs manual UKA where the rUKA group was revision-free at 5 years, while 9% (*P* < .001) of the mUKA group required additional surgical intervention, suggesting the potential benefits of this technology in reducing revision UKA procedures.^50^

One concern associated with implementation of rKA is the economics and associated cost of this technology compared with manual techniques. In this study, we compared our health plan insured patients’ perioperative and postoperative costs for robotic to manual cases stratified into UKA and TKA. Total perioperative cost differences were significantly higher when robotic arm–assisted technology was used ($738 and $581 difference for UKA and TKA, respectively), despite the robotic groups having lower mean OR costs than manual groups for both UKA and TKA (**Table 4**). The lion’s share of higher cost differences arose from preoperative CT scan, supplies (primarily robotic-specific consumables used in the OR), and mean recovery room costs. The recovery room charges are time based. We found robotic procedures were commonly lead-off or morning surgeries and had longer recovery room stays due to transfer delays to hospital rooms related to capacity limitations, accounting for the higher recovery room costs.

A significantly larger portion of the manual UKA and TKA patients had inpatient room costs, despite mean LOS in days being approximately equal for all groups. Looking at the absolute LOS range in days, this was significantly greater for rKA patients (rUKA range = 2.9 days vs mUKA range = 1.1; rTKA range = 3.8 days vs mTKA range = 2.2). Although a higher percentage of robotic patients were discharged sooner, the robotic groups had a greater number of long-stay outliers compared with the manual groups, explaining the near-equal mean length of stay.

Robotic patients had significantly fewer home health visits and corresponding mean home health costs tended to be lower, although the latter differences were not significant. Similarly, robotic patients had significantly fewer home rehab visits, and, again, corresponding mean home rehab costs tended to be lower but did not reach significance. There were also no significant differences in outpatient rehab costs. Finally, no significant differences were found with respect to total rehab costs and total postoperative costs, although these tended to be lower on average for rKA.

Our combined mean total perioperative and postoperative costs for manual and robotic UKA and TKA revealed the lowest cost was associated with mUKA ($4526), while rUKA ($5076) was less costly than mTKA ($5689) and significantly less costly than rTKA ($5864). There have been several prior studies on the costs of rTKA compared with mTKA, in contrast to our findings. In 2019, Cool et al^32^ reported the 90-day episode-of-care cost of rTKA incurred an overall lower cost vs mTKA, with saving driven by reduced LOS, fewer readmissions, and beneficial discharge destination. Mont et al^9^ also reported lower 90-day costs for rTKA through decreased postoperative healthcare utilization (home health, emergency room services, and readmissions). Another comparative cost analysis study of rTKA and mTKA revealed higher intraoperative costs associated with rTKA, similar to our findings, but these higher costs were offset by greater savings in postoperative costs for the 90-day episode. These cost savings encompassed reduced instrument processing fees, shorter LOS, fewer prescribed opioids, and fewer patients discharged to SNF, some of which our study did not evaluate.^51^ Two of these studies utilized the Centers for Medicare & Medicaid Services (CMS) administrative database, which is limited in the ability to determine claims costs vs true hospital facility costs.^9,32^ Our study captured 100% of inpatient and outpatient costs for analysis because of the institution’s integrated health insurance plan. Our data revealed rKA was more expensive up to 90 days postoperatively compared with manual procedures for both UKA and TKA by $550 and $176, respectively. Using a time-driven activity-based costing analysis, Fang et al^52^ evaluated only in-hospital costs comparing rTKA with conventional TKA. Overall hospital costs for rTKA were 1.10 times more costly than mTKA, which correlates with our perioperative cost findings. Unfortunately, the episode-of-care cost in this study was limited to the point of hospital discharge and did not include any postdischarge expenses, where other studies identified cost saving in rTKA through reduced postoperative healthcare services utilization.^9,32,53^ Pierce et al^53^ used a commercial claims data to compare rTKA and mTKA and reported shorter LOS, reduced utilization of services, and reduced 90-day payer costs for rTKA. The costs associated with overall postsurgery expenditures were significantly less in rTKA in that study. Our study’s average total postoperative costs were also less for both rTKA and rUKA but did not reach statistical significance. Due to the use of a commercial claims database, cohort in the Pierce et al study was almost 2000 patients larger.^53^ More patients with rKA were discharged the same day in our study, and a similar LOS trend may be observed at our institution with a larger sample size.

Surgeons included in our study performed higher volumes of UKA, and UKA comprised a greater percentage of total surgical volume after the introduction of robotic-assisted technology. This increased utility of UKA in selected patients has the potential to reduce overall KA cost by supplanting a portion of costlier TKA volume, as the average per-case cost of rUKA was less than that of manual and robotic TKA by $613 and $788, respectively, in our study. Others have suggested UKA provides a cost-effective alternative to TKA in appropriately selected patients.^54^ According to Burn et al,^55^ UKA can be expected to generate better health outcomes and lower lifetime costs than TKA when performed by high-volume surgeons.

Our integrated health system’s insurance plan database allowed 100% of inpatient and outpatient expenses to be captured for cost analysis. This complete and granular level of financial data is a major strength of this study. Yet this study must be interpreted in light of important limitations. First, this is a retrospective study and subject to the deficiencies of retrospective design, including the limitations of establishing well-defined matched study groups. Our patient groups are relatively small, in which case the mean values in the data may be influenced by outliers. The study group was restricted by exclusion of low-volume surgeons, those who did not use the robotic arm–assisted technology, or who were hired or departed the health system during the study period. Another factor limiting study group size was that only patients with our affiliated institutional insurance plan were included for cost analyses. This particular restriction also served as a strength of our study by allowing complete capture of all inpatient and outpatient cost data for each case, although not all costs categories were included in our analysis. There was no defined process by which surgeons decided which patients received rKA. This decision was at the individual surgeon’s discretion, thus lending to possible selection bias. This study includes surgical data from 4 established surgeons and 2 hospitals utilizing uniform postoperative protocols and order sets but is not representative of all healthcare systems or surgical practices. It is important to note that our study does not and could not quantify the economic advantage a hospital may realize by offering rKA. We also do not have a way to quantify or account for better patient-reported outcomes and higher patient satisfaction experienced by rKA patients, which several studies have demonstrated.^8–18^ This study only includes cost data through the 90-day postoperative period. Finally, we did not include the capital expenditure of institutional investment in the robotic technology.

## CONCLUSIONS

This study shows the introduction of rKA technology resulted in an increased incidence and volume of UKA procedures performed. Cost analysis indicates rKA is less expensive in several cost categories but more expensive overall for both UKA and TKA when compared with manual techniques in our system. Longer-term outcomes and cost data are necessary to evaluate the economic impact of rKA. As medical technology emerges, cost-effectiveness and fiscal research analyses are essential to legitimize investments, formulating the concept of “evidence-based healthcare spending.” This type of analysis will help optimize costs in the future.

### Disclosures

D.S.H., R.T.R., J.L.H., and D.J.K. declare no conflicts of interest. J.H.G. has received funding unrelated to this work from Medtronic, Pfizer Inc, Purdue Pharma LP, and Astra Zeneca.

### Author Contributions

D.J.K. and J.L.H. conceived the study and participated in its design and coordination. D.S.H., R.T.R., and J.L.H. participated in the data capture and analysis. D.J.K., D.S.H., J.L.H., R.T.R., and J.H.G. participated in the manuscript drafting and review. J.H.G. performed the statistical analysis.
